# MEDTEC Students against Coronavirus: Investigating the Role of Hemostatic Genes in the Predisposition to COVID-19 Severity

**DOI:** 10.3390/jpm11111166

**Published:** 2021-11-09

**Authors:** Claudio Cappadona, Elvezia Maria Paraboschi, Nicole Ziliotto, Sandro Bottaro, Valeria Rimoldi, Alessio Gerussi, Andrea Azimonti, Daniele Brenna, Andrea Brunati, Charlotte Cameroni, Giovanni Campanaro, Francesca Carloni, Giacomo Cavadini, Martina Ciravegna, Antonio Composto, Giuseppe Converso, Pierluigi Corbella, Davide D’Eugenio, Giovanna Dal Rì, Sofia Maria Di Giorgio, Maria Chiara Grondelli, Lorenza Guerrera, Georges Laffoucriere, Beatrice Lando, Leandro Lopedote, Benedetta Maizza, Elettra Marconi, Carlotta Mariola, Guia Margherita Matronola, Luca Maria Menga, Giulia Montorsi, Antonio Papatolo, Riccardo Patti, Lorenzo Profeta, Vera Rebasti, Alice Smidili, Sofia Maria Tarchi, Francesco Carlo Tartaglia, Gaia Tettamanzi, Elena Tinelli, Riccardo Stuani, Cristiana Bolchini, Linda Pattini, Pietro Invernizzi, Frauke Degenhardt, Andre Franke, Stefano Duga, Rosanna Asselta

**Affiliations:** 1 Department of Biomedical Sciences, Humanitas University, Via Rita Levi Montalcini 4, 20072 Pieve Emanuele, Italy; claudio.cappadona@humanitasresearch.it (C.C.); elvezia_maria.paraboschi@hunimed.eu (E.M.P.); nicole.ziliotto@humanitasresearch.it (N.Z.); sandro.bottaro@hunimed.eu (S.B.); valeria.rimoldi@hunimed.eu (V.R.); andrea.azimonti@st.hunimed.eu (A.A.); daniele.brenna@st.hunimed.eu (D.B.); andrea.brunati@st.hunimed.eu (A.B.); charlotte.cameroni@st.hunimed.eu (C.C.); giovanni.campanaro@st.hunimed.eu (G.C.); francesca.carloni@st.hunimed.eu (F.C.); giacomo.cavadini@st.hunimed.eu (G.C.); martina.ciravegna@st.hunimed.eu (M.C.); antonio.composto@st.hunimed.eu (A.C.); giuseppe.converso@st.hunimed.eu (G.C.); pierluigi.corbella@st.hunimed.eu (P.C.); davide.deugenio@st.hunimed.eu (D.D.); giovanna.dalri@st.hunimed.eu (G.D.R.); sofia.digiorgio@st.hunimed.eu (S.M.D.G.); mariachiara.grondelli@st.hunimed.eu (M.C.G.); lorenza.guerrera@st.hunimed.eu (L.G.); georges.laffoucriere@st.hunimed.eu (G.L.); beatrice.lando@st.hunimed.eu (B.L.); leandro.lopedote@st.hunimed.eu (L.L.); benedetta.maizza@st.hunimed.eu (B.M.); elettra.marconi@st.hunimed.eu (E.M.); carlotta.mariola@st.hunimed.eu (C.M.); guiamargherita.matronola@st.hunimed.eu (G.M.M.); lucamaria.menga@st.hunimed.eu (L.M.M.); giulia.montorsi@st.hunimed.eu (G.M.); antonio.papatolo@st.hunimed.eu (A.P.); riccardo.patti@st.hunimed.eu (R.P.); lorenzo.profeta@st.hunimed.eu (L.P.); vera.rebasti@st.hunimed.eu (V.R.); alice.smidili@st.hunimed.eu (A.S.); sofiamaria.tarchi@st.hunimed.eu (S.M.T.); francesco.tartaglia@st.hunimed.eu (F.C.T.); gaia.tettamanzi@st.hunimed.eu (G.T.); elena.tinelli@st.hunimed.eu (E.T.); riccardo.stuani@st.hunimed.eu (R.S.); stefano.duga@hunimed.eu (S.D.); 2 Humanitas Clinical and Research Center, IRCCS, Via Manzoni 56, 20089 Rozzano, Italy; 3 Division of Gastroenterology, Center for Autoimmune Liver Diseases, Department of Medicine and Surgery, University of Milano-Bicocca, 20900 Monza, Italy; a.gerussi@campus.unimib.it (A.G.); pietro.invernizzi@unimib.it (P.I.); 4 European Reference Network on Hepatological Diseases (ERN RARE-LIVER), San Gerardo Hospital, 20900 Monza, Italy; 5 Department of Electronics, Information and Bioengineering, Politecnico di Milano, 20133 Milan, Italy; cristiana.bolchini@polimi.it (C.B.); linda.pattini@polimi.it (L.P.); 6 Institute of Clinical Molecular Biology, Christian-Albrechts-University Kiel, 24105 Kiel, Germany; f.degenhardt@ikmb.uni-kiel.de (F.D.); a.franke@ikmb.uni-kiel.de (A.F.); 7 University Hospital Schleswig-Holstein (UKSH), 24105 Kiel, Germany

**Keywords:** SARS-CoV-2, COVID-19, hemostatic genes, association analysis, polygenic risk score, meta-analysis, *MTHFR*

## Abstract

Severe acute respiratory syndrome coronavirus 2 (SARS-CoV-2) is the etiologic agent of the coronavirus disease 2019 (COVID-19) pandemic. Besides virus intrinsic characteristics, the host genetic makeup is predicted to account for the extreme clinical heterogeneity of the disease, which is characterized, among other manifestations, by a derangement of hemostasis associated with thromboembolic events. To date, large-scale studies confirmed that genetic predisposition plays a role in COVID-19 severity, pinpointing several susceptibility genes, often characterized by immunologic functions. With these premises, we performed an association study of common variants in 32 hemostatic genes with COVID-19 severity. We investigated 49,845 single-nucleotide polymorphism in a cohort of 332 Italian severe COVID-19 patients and 1668 controls from the general population. The study was conducted engaging a class of students attending the second year of the MEDTEC school (a six-year program, held in collaboration between Humanitas University and the Politecnico of Milan, allowing students to gain an MD in Medicine and a Bachelor’s Degree in Biomedical Engineering). Thanks to their willingness to participate in the fight against the pandemic, we evidenced several suggestive hits (*p* < 0.001), involving the *PROC*, *MTHFR*, *MTR*, *ADAMTS13*, and *THBS2* genes (top signal in *PROC*: chr2:127192625:G:A, OR = 2.23, 95%CI = 1.50–3.34, *p* = 8.77 × 10^−5^). The top signals in *PROC*, *MTHFR*, *MTR*, *ADAMTS13* were instrumental for the construction of a polygenic risk score, whose distribution was significantly different between cases and controls (*p* = 1.62 × 10^−8^ for difference in median levels). Finally, a meta-analysis performed using data from the Regeneron database confirmed the contribution of the *MTHFR* variant chr1:11753033:G:A to the predisposition to severe COVID-19 (pooled OR = 1.21, 95%CI = 1.09–1.33, *p* = 4.34 × 10^−14^ in the weighted analysis).

## 1. Introduction

Severe acute respiratory syndrome coronavirus 2 (SARS-CoV-2) is the etiologic agent of the coronavirus disease 2019 (COVID-19) pandemic. Since late 2019, the virus has infected more than 236 million people and claimed more than 4.8 million lives worldwide (October 8, 2021) [1]. Clinical manifestations are extremely heterogeneous, ranging from the absence of symptoms (in the vast majority of infected individuals) to pneumonia with hypoxemia in more severe patients, acute respiratory distress syndrome, sepsis, and multi-organ failure in critical cases [2]. The severe forms of the disease have been consistently associated with age, comorbidities, and male sex [3,4].

Besides intrinsic characteristics of the virus, the genetic makeup of the host likely accounts for the observed clinical heterogeneity of COVID-19 [5]. In this frame, several genome-wide association studies (GWAS) and meta-analyses have shown that genetic predisposition plays a role in COVID-19 severity and susceptibility [6,7,8,9,10,11]. The first reported genome-wide association signals were at the 3p21.31 and 9q34.2 loci, respectively pointing to a cluster of genes (coding for proteins regulating bronchial physiology, viral entry, and immune response) and to the ABO blood group gene. These associations have been repeatedly replicated in subsequent studies, which also evidenced the existence of at least 13 additional genome-wide significant associations with SARS-CoV-2 infection or COVID-19 severity [7,8,9,10,11]. The contribution of rare variants to COVID-19 just started to be elucidated: exome/genome sequencing in 659 patients with life-threatening COVID-19 pneumonia and 534 positive asymptomatics demonstrated that 3.5% of patients had genetic defects at 8 of the 13 loci involved in inborn errors of type I interferon (IFN) immunity [12]. These results were corroborated by the observation that phenocopies of inborn errors of type I IFN immunity account for severe COVID-19 pneumonia in 2.6% of women and 12.5% of men [13]. On the other hand, Povysil and colleagues [14] performed a large, multi-country sequencing study on patients with mild and severe COVID-19 and observed no enrichment of loss-of-function variants in genes in the type I IFN pathway. Hence, notwithstanding this growing body of information, the genetic host factors underpinning the acute responses in severe COVID-19 patients are still largely to be disclosed.

COVID-19 pathogenesis has been associated with a derangement of hemostasis and with thromboembolic events. Specifically, in the early phases of the disease course, increased D-dimer levels, which are indicators of fibrinolysis and coagulopathy, are a predictive biomarker of both severe disease and lethality in COVID-19 patients [15]. Low platelet count (an indicator of abnormally increased activation of the procoagulant pathway) has been registered in 20% of patients who died in hospital compared to 1% of those who recovered [15]. It also seems that systemic activity of coagulation factors V (FV), VIII (FVIII), and X (FX) are abnormally increased in severe COVID-19 patients [16]. More in general, COVID-19 patients face an elevated risk of pulmonary embolism and venous, arterial, and microvascular thrombosis [17,18,19,20]. The molecular mechanisms linking coronavirus infection and deregulation of hemostasis are not yet well understood, but plausible hypotheses have been suggested, especially considering the connection between hemostasis and inflammation. Indeed, acute lung injury deriving from viral cytopathic effects, the activation of the complement cascade, and the COVID-19-associated cytokine storm have all been proposed to activate the coagulation cascade [21,22].

With these premises, in this study we analyzed the association of common variants in hemostatic genes with COVID-19 severity. Considering the tremendous impact that the pandemic had not only on patients but also on the entire society and the consequent pervasive desire to participate, at any level, in the fight against the disease, we decided to engage our students attending the second year of the medical school in this research project. In particular, a class of students following the MEDTEC school (an integrated six-year training program that allows students to obtain an MD in Medicine as well as a Bachelor’s Degree in Biomedical Engineering) were asked to participate to the analyses in the frame of the practical activities of the course in “Molecular and Computational Biology and Medical Genetics”.

## 2. Materials and Methods

### 2.1. Patient Cohorts for Genetic Analyses

For association analyses, we investigated: (i) 347 patients with severe COVID-19, which was defined as hospitalization with respiratory failure and a confirmed SARS-CoV-2 viral RNA PCR test from nasopharyngeal swabs. Patients were recruited from intensive care units and general wards at two hospitals in the Milan area, i.e., the Humanitas Clinical and Research Center, IRCCS, in Rozzano (140 patients); and the San Gerardo Hospital, in Monza (192 patients); (ii) 1696 controls from the general Italian population with unknown COVID-19 status.

Details on DNA extraction, array genotyping and quality checks are reported elsewhere [6,23].

### 2.2. Imputation

Genetic coverage was increased in the cohort by performing single-nucleotide polymorphism (SNP) imputation on the genome build GRCh38 by using the Michigan Imputation Server [24] and haplotypes generated by the Trans-Omics for Precision Medicine (TOPMed) program (freeze 5) [25]. In the imputation step, we applied the server options to filter by an imputation of R^2^ > 0.3 and to perform a QC frequency check against the TopMed panel. In the post-imputation stage, we only retained those SNPs with R^2^ ≥ 0.6 and minor allele frequency (MAF) ≥ 1%. Next, we accurately checked cases and controls for solving within-Italian relationships and for excluding the possible existence of population stratification across and within batches: to do this, we performed principal component analysis (PCA), using a LD-pruned subset of SNPs across the entire genome and the PLINK v.1.9 package [26]. The final set of variants was composed of 8.6 million SNPs shared across cases and controls.

### 2.3. SNP Selection

A total of 32 hemostatic genes were selected from the literature (Table 1), based on previous evidence of implication in coronary artery disease/myocardial infarction and/or venous thromboembolism susceptibility. For each gene, we considered a genomic region comprising 250 kb upstream and 250 kb downstream the transcriptional unit and extracted from the dataset all the SNPs by using PLINK v.1.9. The final set of variants to be submitted to association analyses comprised 49,845 SNPs.

### 2.4. Statistical Analysis

Case-control allele-dose association tests were performed using the PLINK v.1.9 software. Analyses without the inclusion of covariates in the model (uncorrected) were performed using the *--assoc* and *--ci 0.95* commands. Analyses including the covariates in the model (corrected) made use of a logistic-regression framework for dosage data with the commands *--logistic*, *--covar* and *--ci 0.95*. Age, sex, age*age, sex*age, and the first 10 principal components from PCA were comprised as covariates. For SNPs mapping on the X chromosome, we assumed uniform and complete X-inactivation in females and a similar effect size between males and females (i.e., females are considered to have 0, 1, or 2 copies of an allele, whereas males are considered to have 0 or 2 copies of the same allele). For X chromosome analysis only, we analyzed each sex separately (cases vs. controls). Female-only and male-only *p* values were then combined using the Stouffer’s method [27], which accounts for potential differential effect size and direction between males and females, and also weights the two test statistics (by using the square-root of the male/female sample size). This analysis was performed with XWAS v.3.0 using the commands *--xwas*, *--strat-sex*, *--stouffers*, *--logistic*, *--xchr-model 2*, and *--ci 0.95* [28].

All analyses were conducted referring to the minor allele. *p*-values were not corrected for multiple testing unless otherwise specified and are reported together with odds ratios (OR) and their 95% confidence intervals (CI). We considered loci with *p* < 0.001 as suggestive of association, following the guidelines of Lander and Kruglyak for large-scan association studies [29].

### 2.5. Polygenic Risk Score (PRS)

The PRS was calculated following the classic “clumping + thresholding” (C + T) method, where SNP clumping and a GWAS *p*-value thresholding are performed to control for linkage disequilibrium (LD) and adjust GWAS estimated effect sizes, respectively [30]. The sum of risk alleles of an individual was calculated using the risk allele effect size estimates from an independent GWAS study on the same phenotype. The summary statistics from the COVID-19 Host Genetic Initiative (HGI) [31] GWAS meta-analyses Release 5 dataset were used as reference data to obtain the effect size estimates. The selected dataset (COVID19_HGI_A2_ALL_eur_leave_ukbb_23andme_20210107.txt) contains the results relative to the “Very severe respiratory confirmed covid vs. population” phenotype.

Based on the results obtained in our association analysis, we calculated the PRS using the top variants for each candidate gene filtered with a threshold of association of *p* < 0.001 (corrected values), for a total of 5 variants. Through this step the effect size estimate of all the excluded variants is zero, while the effect size estimate of the retained variants will not be shrunken. The independence of the variants was confirmed through clumping using PLINK 1.9. The PRS was then calculated using PLINK 1.9 *--score* command and *--sum* option, assuming an additive model of independent variants. All the combinations of the 5 selected variants were tested: the PRS explaining the highest phenotypic variance was identified by using the R program [32], by testing the association between the PRS and the severe COVID phenotype in our dataset using a logistic regression. Covariates and confounders were included in the model.

### 2.6. Meta-Analysis

For the meta-analysis step, we retrieved association data deposited in the Regeneron–Genetic Center database [33] from the GHS study (Geisinger Health System; data were available for 869 cases and 112,862 controls of European ancestry). Pooled ORs and CIs were calculated both using the Mantel-Haenszel model [34] and the Fisher weighted method [35,36], in this last case to take into account the imbalance in the number of cases and controls characterizing the two datasets.

### 2.7. Role of MEDTEC Students

MEDTEC students were in charge of extracting SNPs from the imputed dataset and of performing single-SNP association analyses (uncorrected and corrected). Each student was assigned one gene, but they worked in small collaborative groups, double checking the respective analyses.

## 3. Results

### 3.1. Hemostatic Gene Variants and the Predisposition to COVID-19 Severity

To explore the possible role of variants mapping within or in proximity (±250 kb) of 32 hemostatic genes in the predisposition to COVID-19 severity, we performed a single-SNP association analysis between 49,845 genotyped/imputed SNPs and severe COVID-19 with respiratory failure in an Italian cohort of 332 cases and 1668 controls from the general population (post quality-checked cohort).

**Figure 1:**
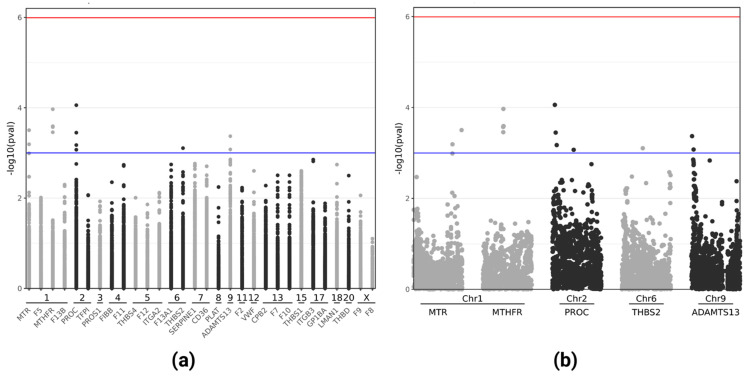
Manhattan plots for the analyzed regions. Manhattan plots summarizing the results of single-gene association analyses (**a**), with a close-up view relative to the top 5 genes (**b**). The horizontal lines represent the suggestive *p* = 0.001 (blue) and the Bonferroni-corrected *p* = 1.02 × 10^−6^ (red) significance levels. Genomic regions belonging to the same chromosome are all represented with the same color (either gray or black). Plots were produced using R.

Table 2 lists the best signals found for each analyzed region (having at least a *p* < 0.05), whereas Figure 1 shows the Manhattan plots visually summarizing the single-gene association analysis. In detail, SNP association analysis revealed several suggestive hits (*p* < 0.001), with the top signal in the *PROC* region (chr2:127192625:G:A, OR = 2.23, 95%CI = 1.50–3.34, *p* = 8.77 × 10^−5^ in the corrected analysis) and additional suggestive associations involving *MTHFR*, *MTR*, *ADAMTS13*, and *THBS2* (Table 2; Figure 1).

Table 3 shows association results for genetic variants known to have a functional impact on the corresponding gene product and to be associated with venous/arterial thrombosis, i.e.,: (i) the Leiden mutation in the *F5* gene (rs6025; p.Arg506Gln); (ii) the variant located in the 3′ untranslated region of the prothrombin gene (known as G20210A, rs1799963); and (iii) the missense variant p.Val34Leu in the *F13A1* gene (rs5985) [37]. We did find only an unexpected weak protective signal with the prothrombin variant (OR = 0.23, 95%CI = 0.070–0.75, *p* = 0.015; Table 3).

### 3.2. Set up of a Poligenic Risk Score Based on Hemostatic Gene Variants

**Figure 2:**
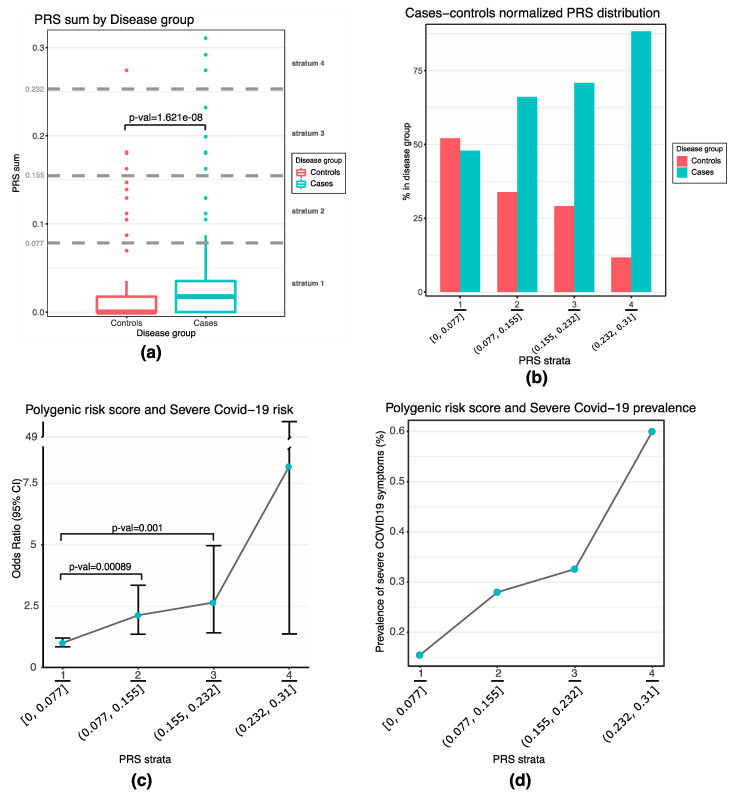
Distribution of the PRS in cases and controls and severe COVID-19 risk in the Italian population. (**a**) Comparison of PRS scores between cases and controls. In the boxplot, boxes define the interquartile range; thick lines refer to the median. Samples were divided into 4 strata (dividing the entire range of PRS values into 4 evenly spaced intervals). Dotted grey lines represent boundaries between each PRS stratum. The *p* value was calculated using the Wilcoxon rank sum test. (**b**) Proportion of cases and controls in each PRS stratum (setting as 100% the overall amount of individuals for each stratum). (**c**) Risk of severe COVID-19, expressed as OR ± 95%CI by strata. ORs were calculated comparing the lowest stratum as reference against all the others. *p*-values from Chi-square test are reported for 2nd and 3rd PRS strata (not indicated for highest stratum due to lower number of individuals). (**d**) Percentage of case samples within each PRS stratum (with 100% as the total number of cases and controls populating that stratum). All plots were produced using R.

Though above the conservative threshold for multiple testing (Bonferroni threshold of significance: *p* = 1 × 10^−6^), single-gene association results encouraged us to evaluate the joint effect of the 5 top variants (*p* < 0.001) mapping in the *PROC*, *MTHFR*, *MTR*, *ADAMTS13*, and *THBS2* regions by calculating a weighted PRS, based on risk allele effect size estimates taken from the COVID-19 HGI dataset (Release 5) [31]. The most significant combination of SNPs was the one ignoring the chr6:169195156:A:T polymorphism (located in *THBS2*), which was hence excluded from further calculations. Figure 2a shows the overall PRS content in severe COVID-19 patients vs. controls: we observed significantly higher PRS values in the case cohort, with median PRS values of 0.017 in cases vs. 0 in controls (*p* = 1.62 × 10^−8^, Wilcoxon rank sum test). Categorizing the PRS of cases and controls into four equally distributed strata, the resulting normalized distributions showed opposite shapes, with decreasing percentages of control individuals in correspondence of higher PRS values, and vice versa for COVID-19 cases (Figure 2b). In particular, the risk of severe COVID-19 increased across the strata of the PRS, with individuals in the second stratum having an OR of 2.12 (95%CI = 1.35–3.35, *p* = 8.9 × 10^−4^), compared with those of the first one (Figure 2c,d). Higher OR values could be observed also for persons belonging to the third and fourth strata (up to OR = 8.19), but the overall number of individuals populating these strata is low, making less confident OR estimates.

### 3.3. Meta-Analysis Confirms the Involvement of MTHFR in Severe COVID-19 Predisposition

Association data for the four leading SNPs mapping in the *PROC*, *MTHFR*, *MTR*, and *ADAMTS13* genes were retrieved from the Regeneron–Genetic Center database. Data referred to a number of genotyped/imputed individuals varying from ≈430 thousands up to ≈898 thousands, depending on the analyzed variant (Table 4). In the meta-analysis, we confirmed the contribution of the *MTHFR* variant chr1:11753033:G:A to the predisposition to severe COVID-19, with a pooled OR of 1.21 (95%CI = 1.09–1.33) and the notable *p* = 4.34 × 10^−14^ in the weighted analysis (Table 4).

## 4. Discussion

In this work, we aimed at evaluating the impact of genetic variation in genes of the hemostatic pathway on the predisposition to severe COVID-19 by analyzing almost 50 thousand common variants distributed across 32 genes. This was made possible also thanks to the collaboration and the great enthusiasm of the group of students of the 2nd year of the MEDTEC program (Humanitas University and Politecnico di Milano), who were eager to give their contribution to the fight against the pandemic, in any possible way. Each one of them was hence made responsible for the analysis of one gene and for double-checking the analyses of their peers.

All genes (with the only exception of *F8*) were nominally associated (*p* < 0.05) with severe COVID-19, even after classic covariate correction. The most convincing evidence for association with severe COVID-19 was found for five loci: two folate metabolic genes (*MTHFR* and *MTR*), two hemostasis inhibitor genes (*PROC* and *ADAMTS13*), and a thrombospondin gene (*THBS2*). None of these signals, though suggestive, survived the Bonferroni threshold for multiple testing. In this frame, we have to underline that such signals emerged notwithstanding the fact that 4 out of our 5 top variants are characterized by a low frequency in the Italian general population (MAF < 4%), and that simple regression models are underpowered for such kind of variants. In addition, the Bonferroni threshold is based on independence of performed tests, but this assumption is not respected in our analyses, given the correlation between tests for most SNPs due to the presence of LD. A straightforward method to take into account the multiple testing issue in large-scale studies is to rely on the haplotype structure of the genome [38,39]. Just as an example, the 550-kb-long region containing the fibrinogen cluster comprised a total of 1606 SNPs (corresponding to a multiple-testing threshold of 3.11 × 10^−5^); this region is indeed characterized by the presence of only three huge -and strong- LD blocks (as it can be observed by using the locus browser page of the GTEx portal) [40], making the calculated Bonferroni threshold definitively too conservative.

In any case, our best associations were corroborated from one hand by the PRS, and from the other by the meta-analysis results. In particular, the PRS distribution, built using the risk allele effect size estimates of the HGI GWAS meta-analyses Release 5 dataset, was significantly different between cases and controls, with subjects in the second stratum having a 2.12-fold increased risk for severe COVID-19 compared to those in the lowest stratum. Concerning the meta-analysis, performed by exploiting the high statistical power available through the Regeneron data, it was possible to specifically highlight a strong signal for the *MTHFR* gene, in correspondence of the chr1:11753033:G:A variant (also known as rs17875978, OR = 1.21, 95%CI = 1.09–1.33, *p* = 4.34 × 10^−14^). The functional impact of the rs17875978 variant is unknown, however, some speculation can be put forward. This variant is located 32 kb downstream from the 3′UTR of the *MTHFR* gene and it tags a region of LD (chr1:11692083-11764076) including a series of whole-blood specific expression quantitative trait loci (eQTLs) [40]. In addition, this region is characterized by the presence of two distal cis enhancer regions (namely, EH38E1319076-EH38E1319075) potentially bound by transcription factors ZNF384, ZNF460, ZNF135, and GFI1B and targeting *MTHFR*. Overall, these observations point to this region as potentially implicated in the regulation of the *MTHFR* gene. More interestingly, a phenome-wide association study (PheWAS) using the broad list of phenotypes from the NHGRI GWAS Catalog [41] while revealing no known trait to be significantly associated with the rs17875978 lead SNP, highlighted a total of nine genome-wide significant (*p* < 5.0 × 10^−8^) associations with the *MTHFR* gene [42,43,44,45,46]. These comprised phenotypes potentially related to COVID-19 pathophysiology, such as high blood pressure, agents acting on the renin-angiotensin system, and mean corpuscular hemoglobin. Indeed, the *MTHFR* gene codes for the 5,10-methylenetetrahydrofolate reductase involved in folate metabolism, essential for the synthesis of nucleotides and single-carbon groups. The MTHFR enzyme converts 5,10-methylenetetrahydrofolate to 5-methyltetrahydrofolate, which produces methyl donors to convert homocysteine to methionine. It has been recently shown that SARS-CoV-2 remodels both the host folate and the one-carbon metabolism at the post-transcriptional level to meet the demand for viral subgenomic RNA replication, bypassing viral shutoff of host translation [47]. Since folate is depleted in SARS-CoV-2- infected cells [47], homocysteine levels should increase and eventually lead to hyper-homocysteinemia, a known risk factor for a variety of complex disorders including cardiovascular and neurological diseases [48], possibly contributing to a severe course of COVID-19 [49]. Interestingly, *MTHFR* variants and homocysteine levels have been suggested as modulators of the risk of COVID-19 incidence and severity [50,51]. Moreover, elevated homocysteine levels may in turn increase tissue factor activity [52,53], thus promoting a prothrombotic state.

In addition to increased levels of pro-coagulant factors, thrombotic complications reported in COVID-19 patients may also arise for impaired endogenous anticoagulants, including Protein C and ADAMTS13. Decreased levels of protein C, a main inhibitor of coagulation, were in fact associated with disease worsening and mortality [54,55], though conflicting data were also reported in smaller patient cohorts [56,57,58]. In-vitro evidence suggested that synthesis and secretion of ADAMTS13 are inhibited by inflammatory cytokines released during systemic inflammation [59]. This might also stimulate the release and inhibit the cleavage of the von Willebrand Factor (vWF) [60], the substrate of the ADAMTS13 protease and the key protein in primary hemostasis (platelet aggregation). Several authors reported hemostatic alterations in vWF/ADAMTS13 axis that were strongly associated with disease severity in COVID-19 patients, including increased levels of vWF [61,62,63,64] accompanied by low normal or mildly decreased ADAMTS13 activity [65,66,67,68,69,70]. Based on these observations, the vWF/ADAMTS13 imbalance has been proposed to be causally related both to the prothrombotic tendency and to the microvascular pulmonary thrombosis, which subtend to a form of thrombotic microangiopathy (TMA) observed in severe COVID-19 patients. The severe deficiency of ADAMTS13 has also gained attention due to the development of a rare TMA, called immune-mediated thrombotic thrombocytopenic purpura [71,72], caused by the production of anti-ADAMTS13 antibodies, in a limited number of subjects after COVID-19 vaccination. Interestingly, vWF significantly increases with age and in non-0 blood type individuals [73], both risk factors for COVID-19.

## 5. Conclusions

In conclusion, though several mechanisms can explain the relationship between viral infection and thrombosis, our study suggests a combined effect of some risk variants mapping in the proximity of genes specifically involved in the regulation of hemostasis on the etiopathology of COVID-19.

## Figures and Tables

**Table 1:** Hemostatic gene loci tested for association with severe COVID-19.

Chr	Gene	Protein	Gene Size (kb)	Analyzed Region (kb)	Coordinates of the Analyzed Region (hg38)	Analyzed SNPs (n)
1	*MTHFR*	MTHR	20.3	520.3	1:11535729-12056103	1821
1	*F5*	FA5 (FV)	74.5	574.5	1:169261953-169836531	2276
1	*F13B*	F13B (FXIII)	28.0	528	1:196789190-197317267	956
1	*MTR*	METH	108.7	608.7	1:236545280-237153981	2270
2	*TFPI*	TFPI	90.2	590.2	2:187214230-187804492	1655
2	*PROC*	PROC	10.8	510.8	2:127168419-127679246	1492
3	*PROS1*	PROS	101.0	601	3:93623036-94224090	592
4	*FGB*	FIBB	49.7	549.7	4:154312979-154862750	1606
4	*FGA*					
4	*FGG*					
4	*F11*	FA11 (FXI)	23.7	523.7	4:186015963-186539681	2251
5	*ITGA2*	ITA2 (GPIa)	105.4	605.4	5:52739325-53344779	2469
5	*THBS4*	TSP4	47.9	547.9	5:79785348-80333284	2047
5	*F12*	FA12 (FXII)	7.4	507.4	5:177152137-177659576	890
6	*F13A1*	F13A (FXIII)	176.6	676.6	6:5894077-6570691	2895
6	*THBS2*	TSP2	38.3	538.3	6:168965779-169504114	2080
7	*CD36*	CD36 (GPIIIb)	304.8	804.8	7:80119575-80924418	2110
7	*SERPINE1*	PAI-1	12.1	512.1	7:100877088-101389266	1929
8	*PLAT*	TPA	32.9	532.9	8:41924717-42457676	927
9	*ADAMTS13*	ATS13 (ADAMTS13)	37.4	537.4	9:133171999-133709403	2195
11	*F2*	THRB	20.3	520.3	11:46469192-46989506	697
12	*VWF*	VWF	175.7	675.7	12:5698873-6374670	2122
13	*CPB2*	CBPB2 (TAFI)	51.8	551.8	13:45803186-46355076	1811
13	*F7*	FA7 (FVII)	14.8	514.8	13:112855787-113370681	1411
13	*F10*	FA10 (FX)	26.7	526.7	13:112872798-113399529	1540
15	*THBS1*	TSP1	17.8	517.8	15:39331078-39848921	1443
17	*GPIBA*	GPIBA	2.7	502.7	17:4682274-5185030	1502
17	*ITGB3*	ITB3 (GPIIIa)	58.8	558.8	17:47003841-47562711	1695
18	*LMAN1*	LMAN1	31.4	531.4	18:59077823-59609276	2327
20	*THBD*	TRBM	4.0	504	20:22795632-23299664	1488
X	*F8*	FA8 (FVIII)	186.9	686.9	23:154585788-155272723	570
X	*F9*	FA9 (FIX)	32.7	532.7	23:139280735-139813458	778
Total	32 genes	-	1893	16,893	-	49,845

Genomic coordinates are based on the genome build GRCh38. *FGA*, *FGB*, and *FGG*, coding for the three subunits of the fibrinogen molecule, are present in cluster on chromosome 4; in this case, we considered for the analysis those SNPs mapping from 250 kb upstream to 250 kb downstream the cluster region. Chr = Chromosome.

**Table 2:** (a) Top association results in selected autosomal genes; (b) Top association results in selected X-chromosome genes.

(a)									
						Uncorrected Analyses		Corrected Analyses	
Chr	Gene	SNP		MAF Cases	MAF Controls	*p* Value	OR (95%CI)	*p* Value	OR (95%CI)
2	*PROC*	chr2:127192625:G:A		0.065	0.037	8.51 × 10^−4^	1.82 (1.28–2.61)	8.77 × 10^−5^	2.23 (1.50–3.34)
1	*MTHFR*	chr1:11753033:G:A		0.247	0.183	1.33 × 10^−4^	1.47 (1.20–1.79)	1.08 × 10^−4^	1.56 (1.25–1.95)
1	*MTR*	chr1:237145686:A:G		0.023	0.010	4.53 × 10^−3^	2.39 (1.29–4.43)	3.14 × 10^−4^	3.49 (1.77–6.88)
9	*ADAMTS13*	chr9:133179750:G:C		0.030	0.011	1.58 × 10^−4^	2.77 (1.60–4.80)	4.26 × 10^−4^	2.90 (1.60–5.24)
6	*THBS2*	chr6:169195156:A:T		0.039	0.021	6.25 × 10^−3^	1.87 (1.19–2.96)	7.84 × 10^−4^	2.35 (1.43–3.87)
17	*ITGB3*	chr17:47019591:G:C		0.321	0.270	7.05 × 10^−3^	1.28 (1.07–1.53)	1.41 × 10^−3^	1.39 (1.14–1.71)
7	*SERPINE1*	chr7:101070945:A:G		0.324	0.267	2.55 × 10^−3^	1.32 (1.10–1.58)	1.73 × 10^−3^	1.38 (1.13–1.69)
6	*F13A1*	chr6:6163858:G:C		0.108	0.070	6.99 × 10^−4^	1.61 (1.22–2.13)	1.80 × 10^−3^	1.67 (1.21–2.31)
18	*LMAN1*	chr18:59208206:A:G		0.401	0.337	1.57 × 10^−3^	1.32 (1.11–1.56)	1.81 × 10^−3^	1.36 (1.12–1.65)
4	*F11*	chr4:186056516:A:G		0.065	0.106	1.31 × 10^−3^	0.59 (0.42–0.82)	1.84 × 10^−3^	0.56 (0.39–0.81)
7	*CD36*	chr7:80591832:AAATCAGC:A		0.039	0.021	6.25 × 10^−3^	1.87 (1.19–2.96)	1.98 × 10^−3^	2.15 (1.33–3.50)
15	*THBS1*	chr15:39455553:G:C		0.178	0.133	2.17 × 10^−3^	1.42 (1.13–1.77)	2.50 × 10^−3^	1.46 (1.14–1.86)
12	*VWF*	chr12:6068637:T:C		0.054	0.031	3.71 × 10^−3^	1.76 (1.20–2.60)	2.50 × 10^−3^	1.95 (1.27–3.01)
13	*F7–F10*	chr13:113257337:C:T		0.288	0.234	3.50 × 10^−3^	1.32 (1.10–1.59)	3.13 × 10^−3^	1.37 (1.11–1.69)
20	*THBD*	chr20:22949512:A:G		0.048	0.025	1.51 × 10^−3^	1.94 (1.28–2.93)	3.18 × 10^−3^	2.01 (1.26–3.19)
4	FG_genes	chr4:154774926:G:A		0.054	0.033	8.81 × 10^−3^	1.67 (1.13–2.45)	4.45 × 10^−3^	1.88 (1.22–2.91)
1	*F13B*	chr1:196913749:A:AT		0.036	0.017	1.84 × 10^−3^	2.12 (1.31–3.44)	5.04 × 10^−3^	2.12 (1.25–3.59)
13	*CPB2*	chr13:45931697:A:G		0.008	0.026	3.59 × 10^−3^	0.28 (0.12–0.70)	5.29 × 10^−3^	0.18 (0.056–0.61)
8	*PLAT*	chr8:42278531:C:T		0.017	0.007	1.79 × 10^−2^	2.33 (1.13–4.77)	5.69 × 10^−3^	2.88 (1.36–6.08)
5	*ITGA2*	chr5:53162220:CAGAG:C		0.029	0.013	4.56 × 10^−3^	2.15 (1.25–3.71)	7.60 × 10^−3^	2.30 (1.25–4.23)
2	*TFPI*	chr2:187303697:A:G		0.030	0.013	1.13 × 10^−3^	2.38 (1.39–4.07)	8.74 × 10^−3^	2.25 (1.23–4.14)
1	*F5*	chr1:169677905:G:A		0.116	0.151	1.90 × 10^−2^	0.74 (0.57–0.95)	9.74 × 10^−3^	0.68 (0.51–0.91)
5	*THBS4*	chr5:80174425:G:T		0.203	0.163	1.23 × 10^−2^	1.31 (1.06–1.61)	9.83 × 10^−3^	1.37 (1.08–1.73)
3	*PROS1*	chr3:94043799:A:C		0.032	0.014	1.84 × 10^−3^	2.24 (1.33–3.76)	1.19 × 10^−2^	2.10 (1.18–3.74)
11	*F2*	chr11:46517560:T:C		0.331	0.382	1.39 × 10^−2^	0.80 (0.63–0.96)	1.19 × 10^−2^	0.77 (0.63–0.95)
17	*GP1BA*	chr17:4921551:T:C		0.020	0.010	2.59 × 10^−2^	2.06 (1.08–3.95)	1.31 × 10^−2^	2.50 (1.21–5.15)
5	*F12*	chr5:177464930:A:G		0.026	0.013	1.71 × 10^−2^	1.97 (1.12–3.46)	1.38 × 10^−2^	2.17 (1.17–4.03)
**(b)**									
**Chr**	**Gene**	**SNP**	***p*** **Value_M**	**OR (95%CI)_M**		***p*** **Value_F**	**OR (95%CI)_F**	**P_Comb_Stouffer**	
X	*F9*	chrX:139407485:G:C	8.5 × 10^−4^	1.27 (0.96–1.68)		0.49	0.91 (0.63–1.31)	0.0046	

Results in Table 2 are presented by ordering variants according to the significance of association (*p* values) in the corrected analysis. Age, sex, age × age, sex × age, and the first 10 principal components from the principal component analysis have been used as covariates in the corrected analysis. As for *F7* and *F10*, their top signals coincide (*F7* and *F10* genomic regions partially overlap). The major/minor alleles are indicated in the name of the variant (chromosome:position:major allele:minor allele). For variants mapping on the X chromosome, males (M) and females (F) were analyzed separately, and the combined *p* value of association has been calculated using the Stouffer’s method (see Materials and Methods). SNPs showing a suggestive association with COVID-19 severity (*p* < 0.001) are highlighted in grey. Bonferroni threshold of significance: *p* = 1 × 10^−6^. Chr = chromosome; CI = confidence interval; MAF = minor allele frequency; OR = odds ratio; SNP = single-nucleotide polymorphism.

**Table 3:** Association results for functional SNPs in *F5*, *F2*, and *F13A1*.

					Corrected Analyses	
Chr	Gene	SNP	SNP ID	Minor Allele	*p* Value	OR (95%CI)
1	*F5*	chr1:169549811:C:T	rs6025	T	0.92	0.96 (0.48–1.94)
6	*F13A1*	chr6:6318562:C:A	rs5985	A	0.91	0.99 (0.78–1.24)
11	*F2*	chr11:46739505:G:A	rs1799963	A	0.015	0.23 (0.070–0.75)

Age, sex, age × age, sex × age, and the first 10 principal components from the principal component analysis have been used as covariates for the corrected analysis. Chr = chromosome; CI = confidence interval; ID = identifier; OR = odds ratio; SNP = single-nucleotide polymorphism.

**Table 4:** Meta-analysis results for the four best hits mapping in the *PROC*, *MTHFR*, *MTR*, and *ADAMTS13* genes.

		Italians (Our Cohort)			Regeneron Cohort			Meta-Analysis		
Gene	SNP	Analysed Individuals (n)	*p* Value	OR (95%CI)	Analysed Individuals (n)	*p* Value	OR (95%CI)	Pooled OR (95%CI)	*p* Value (M-H)	*p* Value (F Weighted)
*PROC*	chr2:127192625:G:A	2000	8.51 × 10^−4^	1.82 (1.28–2.61)	429151	0.026	0.71 (0.53–0.96)	0.98 (0.79–1.22)	0.87	0.12
*MTHFR*	chr1:11753033:G:A	2000	1.33 × 10^−4^	1.47 (1.20–1.79)	654056	0.030	1.13 (1.01–1.28)	1.21 (1.09–1.33)	2.55 × 10^−4^	4.34 × 10^−14^
*MTR*	chr1:237145686:A:G	2000	4.53 × 10^−3^	2.39 (1.29–4.43)	898324	0.98	1.00 (0.88–1.14)	1.03 (0.91–1.18)	0.61	0.0027
*ADAMTS13*	chr9:133179750:G:C	2000	1.58 × 10^−4^	2.77 (1.60–4.80)	656078	0.030	0.75 (0.58–0.97)	0.89 (0.72–1.11)	0.33	0.056

Chr = chromosome; CI = confidence interval; F weigh = Fisher weighted meta-analysis; M-H = Mantel-Haenszel meta-analysis; OR = odds ratio; SNP = single-nucleotide polymorphism. The best consistent meta-analysis result is bolded.

## Data Availability

The dataset for cases was submitted to the European Bioinformatics Institute (www.ebi.ac.uk/gwas (accessed on 1 May 2021)) under accession numbers GCST90000255 and GCST90000256, whereas the dataset for controls is deposited in the Genotypes and Phenotypes database (dbGaP; https://www.ncbi.nlm.nih.gov/gap/ (accessed on 1 May 2021)), under the phs000294.v1.p1 accession code.

## References

[1] Johns Hopkins Coronavirus Resource Center. https://coronavirus.jhu.edu/map.html.

[2] Yuki K., Fujiogi M., Koutsogiannaki S. (2020). COVID-19 pathophysiology: A review. Clin. Immunol..

[3] O’Driscoll M., Ribeiro Dos Santos G., Wang L., Cummings D.A.T., Azman A.S., Paireau J., Fontanet A., Cauchemez S., Salje H. (2021). Age-specific mortality and immunity patterns of SARS-CoV-2. Nature.

[4] Williamson E.J., Walker A.J., Bhaskaran K., Bacon S., Bates C., Morton C.E., Curtis H.J., Mehrkar A., Evans D., Inglesby P. (2020). Factors associated with COVID-19-related death using OpenSAFELY. Nature.

[5] Asselta R., Paraboschi E.M., Mantovani A., Duga S. (2020). ACE2 and TMPRSS2 variants and expression as candidates to sex and country differences in COVID-19 severity in Italy. Aging.

[6] Ellinghaus D., Degenhardt F., Bujanda L., Buti M., Albillos A., Invernizzi P., Fernández J., Prati D., Baselli G., Severe COVID-19 GWAS Group (2020). Genomewide Association Study of Severe COVID-19 with Respiratory Failure. N. Engl. J. Med..

[7] Shelton J.F., Shastri A.J., Ye C., Weldon C.H., Filshtein-Sonmez T., Coker D., Symons A., Esparza-Gordillo J., Aslibekyan S., 23andMe COVID-19 Team (2021). Trans-ancestry analysis reveals genetic and nongenetic associations with COVID-19 susceptibility and severity. Nat. Genet..

[8] Pairo-Castineira E., Clohisey S., Klaric L., Bretherick A.D., Rawlik K., Pasko D., Walker S., Parkinson N., Fourman M.H., Russell C.D. (2021). Genetic mechanisms of critical illness in COVID-19. Nature.

[9] Kosmicki J.A., Horowitz J.E., Banerjee N., Lanche R., Marcketta A., Maxwell E., Bai X., Sun D., Backman J., Sharma D. (2020). Genetic association analysis of SARS-CoV-2 infection in 455,838 UK Biobank participants. medRxiv.

[10] Horowitz J.E., Kosmicki J.A., Damask A., Sharma D., Roberts G.H.L., Justice A.E., Banerjee N., Coignet M.V., Yadav A., Leader J.B. (2021). Common genetic variants identify targets for COVID-19 and individuals at high risk of severe disease. medRxiv.

[11] COVID-19 Host Genetics Initiative (2021). Mapping the human genetic architecture of COVID-19. Nature.

[12] Zhang Q., Bastard P., Liu Z., Le Pen J., Moncada-Velez M., Chen J., Ogishi M., Sabli I.K.D., Hodeib S., Korol C. (2020). Inborn errors of type I IFN immunity in patients with life-threatening COVID-19. Science.

[13] Bastard P., Rosen L.B., Zhang Q., Michailidis E., Hoffmann H.H., Zhang Y., Dorgham K., Philippot Q., Rosain J., Béziat V. (2020). Autoantibodies against type I IFNs in patients with life-threatening COVID-19. Science.

[14] Povysil G., Butler-Laporte G., Shang N., Wang C., Khan A., Alaamery M., Nakanishi T., Zhou S., Forgetta V., Eveleigh R.J.M. (2021). Rare loss-of-function variants in type I IFN immunity genes are not associated with severe COVID-19. J. Clin. Investig..

[15] Zhou F., Yu T., Du R., Fan G., Liu Y., Liu Z., Xiang J., Wang Y., Song B., Gu X. (2020). Clinical course and risk factors for mortality of adult inpatients with COVID-19 in Wuhan, China: A retrospective cohort study. Lancet.

[16] Stefely J.A., Christensen B.B., Gogakos T., Cone Sullivan J.K., Montgomery G.G., Barranco J.P., Van Cott E.M. (2020). Marked factor V activity elevation in severe COVID-19 is associated with venous thromboembolism. Am. J. Hematol..

[17] Huang C., Wang Y., Li X., Ren L., Zhao J., Hu Y., Zhang L., Fan G., Xu J., Gu X. (2020). Clinical features of patients infected with 2019 novel coronavirus in Wuhan, China. Lancet.

[18] Kipshidze N., Dangas G., White C.J., Kipshidze N., Siddiqui F., Lattimer C.R., Carter C.A., Fareed J. (2020). Viral Coagulopathy in Patients With COVID-19: Treatment and Care. Clin. Appl. Thromb. Hemost..

[19] McGonagle D., O’Donnell J.S., Sharif K., Emery P., Bridgewood C. (2020). Immune mechanisms of pulmonary intravascular coagulopathy in COVID-19 pneumonia. Lancet Rheumatol..

[20] Tsivgoulis G., Palaiodimou L., Zand R., Lioutas V.A., Krogias C., Katsanos A.H., Shoamanesh A., Sharma V.K., Shahjouei S., Baracchini C. (2020). COVID-19 and cerebrovascular diseases: A comprehensive overview. Ther. Adv. Neurol. Disord..

[21] Merrill J.T., Erkan D., Winakur J., James J.A. (2020). Emerging evidence of a COVID-19 thrombotic syndrome has treatment implications. Nat. Rev. Rheumatol..

[22] Zuo Y., Estes S.K., Ali R.A., Gandhi A.A., Yalavarthi S., Shi H., Sule G., Gockman K., Madison J.A., Zuo M. (2020). Prothrombotic autoantibodies in serum from patients hospitalized with COVID-19. Sci Transl. Med..

[23] Kathiresan S., Voight B.F., Purcell S., Musunuru K., Ardissino D., Mannucci P.M., Anand S., Engert J.C., Samani N.J., Myocardial Infarction Genetics Consortium (2009). Genome-wide association of early-onset myocardial infarction with single nucleotide polymorphisms and copy number variants. Nat. Genet..

[24] TOPMed Imputation Server. https://imputation.biodatacatalyst.nhlbi.nih.gov/index.html#!.

[25] Taliun D., Harris D.N., Kessler M.D., Carlson J., Szpiech Z.A., Torres R., Taliun S.A.G., Corvelo A., Gogarten S.M., Kang H.M. (2021). Sequencing of 53,831 diverse genomes from the NHLBI TOPMed Program. Nature.

[26] Chang C.C., Chow C.C., Tellier L.C., Vattikuti S., Purcell S.M., Lee J.J. (2015). Second-generation PLINK: Rising to the challenge of larger and richer datasets. Gigascience.

[27] Stouffer S.A., Suchman E.A., Devinney L.C., Star S.A., Williams R.M. (1949). Adjustment during Army Life.

[28] Gao F., Chang D., Biddanda A., Ma L., Guo Y., Zhou Z., Keinan A. (2015). XWAS: A Software Toolset for Genetic Data Analysis and Association Studies of the X Chromosome. J. Hered.

[29] Lander E., Kruglyak L. (1995). Genetic dissection of complex traits: Guidelines for interpreting and reporting linkage results. Nat. Genet..

[30] Choi S.W., Mak T.S.H., O’Reilly P.F. (2020). Tutorial: A guide to performing polygenic risk score analyses. Nat. Protoc..

[31] The Covid19 Host Genetics Initiative. https://www.covid19hg.org/.

[32] R Core Team (2017). R: A Language and Environment for Statistical Computing.

[33] The Regeneron–Genetic Center Database. https://rgc-covid19.regeneron.com/home.

[34] Mantel N., Haenszel W. (1959). Statistical aspects of the analysis of data from retrospective studies of disease. J. Natl. Cancer Inst..

[35] Mosteller F., Bush R.R., Lindzey G. (1954). Selected quantitative techniques. Handbook of Social Psychology.

[36] Liptak T. (1958). On the combination of independent tests. Magyar. Tud Akad Mat. Kutato Int. Kozl..

[37] Rosendaal F.R., Reitsma P.H. (2009). Genetics of venous thrombosis. J. Thromb. Haemost..

[38] Nyholt D.R. (2004). A simple correction for multiple testing for single-nucleotide polymorphisms in linkage disequilibrium with each other. Am. J. Hum. Genet..

[39] Nicodemus K.K., Liu W., Chase G.A., Tsai Y.Y., Fallin M.D. (2005). Comparison of type I error for multiple test corrections in large single-nucleotide polymorphism studies using principal components versus haplotype blocking algorithms. BMC Genet..

[40] Genotype-Tissue Expression-GTEx Portal. https://gtexportal.org/home/locusBrowserPage/FGA.

[41] Atlas of GWAS Summary Statistics. https://atlas.ctglab.nl/.

[42] Zhu Z., Wang X., Li X., Lin Y., Shen S., Liu C.L., Hobbs B.D., Hasegawa K., Liang L., International COPD Genetics Consortium (2019). Genetic overlap of chronic obstructive pulmonary disease and cardiovascular disease-related traits: A large-scale genome-wide cross-trait analysis. Respir Res..

[43] Watanabe K., Stringer S., Frei O., Umićević Mirkov M., de Leeuw C., Polderman T.J.C., van der Sluis S., Andreassen O.A., Neale B.M., Posthuma D. (2019). A global overview of pleiotropy and genetic architecture in complex traits. Nat. Genet..

[44] Wu Y., Byrne E.M., Zheng Z., Kemper K.E., Yengo L., Mallett A.J., Yang J., Visscher P.M., Wray N.R. (2019). Genome-wide association study of medication-use and associated disease in the UK Biobank. Nat. Commun..

[45] Astle W.J., Elding H., Jiang T., Allen D., Ruklisa D., Mann A.L., Mead D., Bouman H., Riveros-Mckay F., Kostadima M.A. (2016). The Allelic Landscape of Human Blood Cell Trait Variation and Links to Common Complex Disease. Cell.

[46] International Consortium for Blood Pressure Genome-Wide Association Studies (2011). Genetic variants in novel pathways influence blood pressure and cardiovascular disease risk. Nature.

[47] Zhang Y., Guo R., Kim S.H., Shah H., Zhang S., Liang J.H., Fang Y., Gentili M., Leary C.N.O., Elledge S.J. (2021). SARS-CoV-2 hijacks folate and one-carbon metabolism for viral replication. Nat. Commun..

[48] Sharma P., Senthilkumar R.D., Brahmachari V., Sundaramoorthy E., Mahajan A., Sharma A., Sengupta S. (2006). Mining literature for a comprehensive pathway analysis: A case study for retrieval of homocysteine related genes for genetic and epigenetic studies. Lipids Health Dis..

[49] Karst M., Hollenhorst J., Achenbach J. (2020). Life-threatening course in coronavirus disease 2019 (COVID-19): Is there a link to methylenetetrahydrofolic acid reductase (MTHFR) polymorphism and hyperhomocysteinemia?. Med. Hypotheses.

[50] Ponti G., Pastorino L., Manfredini M., Ozben T., Oliva G., Kaleci S., Iannella R., Tomasi A. (2021). COVID-19 spreading across world correlates with C677T allele of the methylenetetrahydrofolate reductase (MTHFR) gene prevalence. J. Clin. Lab. Anal..

[51] Ponti G., Roli L., Oliva G., Manfredini M., Trenti T., Kaleci S., Iannella R., Balzano B., Coppola A., Fiorentino G. (2021). Homocysteine (Hcy) assessment to predict outcomes of ho-spitalized COVID-19 patients: A multicenter study on 313 Covid-19 patients. Clin. Chem. Lab. Med..

[52] Durand P., Lussier-Cacan S., Blache D. (1997). Acute methionine load-induced hyperhomocysteinemia enhances platelet aggregation, thromboxane biosynthesis, and macrophage-derived tissue factor activity in rats. FASEB J..

[53] Fryer R.H., Wilson B.D., Gubler D.B., Fitzgerald L.A., Rodgers G.M. (1993). Homocysteine, a risk factor for premature vascular disease and thrombosis, induces tissue factor activity in endothelial cells. Arterioscler. Thromb..

[54] Zhang Y., Cao W., Jiang W., Xiao M., Li Y., Tang N., Liu Z., Yan X., Zhao Y., Li T. (2020). Profile of natural anticoagulant, coagulant factor and anti-phospholipid antibody in critically ill COVID-19 patients. J. Thromb. Thrombolysis.

[55] Stanne T.M., Pedersen A., Gisslén M., Jern C. (2021). Low admission protein C levels are a risk factor for disease worsening and mortality in hospitalized patients with COVID-19. Thromb. Res..

[56] Corrêa T.D., Cordioli R.L., Campos Guerra J.C., Caldin da Silva B., Dos Reis Rodrigues R., de Souza G.M., Midega T.D., Campos N.S., Carneiro B.V., Campos F.N.D. (2020). Coagulation profile of COVID-19 patients admitted to the ICU: An exploratory study. PLoS ONE.

[57] Panigada M., Bottino N., Tagliabue P., Grasselli G., Novembrino C., Chantarangkul V., Pesenti A., Peyvandi F., Tripodi A. (2020). Hypercoagulability of COVID-19 patients in intensive care unit: A report of thromboelastography findings and other parameters of hemostasis. J. Thromb. Haemost..

[58] Hardy M., Michaux I., Lessire S., Douxfils J., Dogné J.M., Bareille M., Horlait G., Bulpa P., Chapelle C., Laporte S. (2020). Prothrombotic hemostasis disturbances in patients with severe COVID-19: Individual daily data. Data Brief..

[59] Cao W.J., Niiya M., Zheng X.W., Shang D.Z., Zheng X.L. (2008). Inflammatory cytokines inhibit ADAMTS13 synthesis in hepatic stellate cells and endothelial cells. J. Thromb. Haemost..

[60] Bernardo A., Ball C., Nolasco L., Moake J.F., Dong J.F. (2004). Effects of inflammatory cytokines on the release and cleavage of the endothelial cell-derived ultralarge von Willebrand factor multimers under flow. Blood.

[61] Goshua G., Pine A.B., Meizlish M.L., Chang C.H., Zhang H., Bahel P., Baluha A., Bar N., Bona R.D., Burns A.J. (2020). Endotheliopathy in COVID-19-associated coagulopathy: Evidence from a single-centre, cross-sectional study. Lancet Haematol..

[62] Ward S.E., Curley G.F., Lavin M., Fogarty H., Karampini E., McEvoy N.L., Clarke J., Boylan M., Alalqam R., Worrall A.P. (2021). Von Willebrand factor propeptide in severe coronavirus disease 2019 (COVID-19): Evidence of acute and sustained endothelial cell activation. Br. J. Haematol..

[63] Helms J., Tacquard C., Severac F., Leonard-Lorant I., Ohana M., Delabranche X., Merdji H., Clere-Jehl R., Schenck M., Fagot Gandet F. (2020). High risk of thrombosis in patients with severe SARS-CoV-2 infection: A multicenter prospective cohort study. Intensive Care Med..

[64] Peyvandi F., Artoni A., Novembrino C., Aliberti S., Panigada M., Boscarino M., Gualtierotti R., Rossi F., Palla R., Martinelli I. (2021). Hemostatic alterations in COVID-19. Haematologica.

[65] Ward S.E., Fogarty H., Karampini E., Lavin M., Schneppenheim S., Dittmer R., Morrin H., Glavey S., Ni Cheallaigh C., Bergin C. (2021). ADAMTS13 regulation of VWF multimer distribution in severe COVID-19. J. Thromb. Haemost..

[66] Martinelli N., Montagnana M., Pizzolo F., Friso S., Salvagno G.L., Forni G.L., Gianesin B., Morandi M., Lunardi C., Lippi G. (2020). A relative ADAMTS13 deficiency supports the presence of a secondary microangiopathy in COVID 19. Thromb. Res..

[67] Philippe A., Gendron N., Bory O., Beauvais A., Mirault T., Planquette B., Sanchez O., Diehl J.L., Chocron R., Smadja D.M. (2021). Von Willebrand factor collagen-binding capacity predicts in-hospital mortality in COVID-19 patients: Insight from VWF/ADAMTS13 ratio imbalance. Angiogenesis.

[68] Marco A., Marco P. (2021). Von Willebrand factor and ADAMTS13 activity as clinical severity markers in patients with COVID-19. J. Thromb. Thrombolysis.

[69] Sweeney J.M., Barouqa M., Krause G.J., Gonzalez-Lugo J.D., Rahman S., Gil M.R. (2021). Low ADAMTS13 Activity Correlates with Increased Mortality in COVID-19 Patients. TH Open.

[70] Pascreau T., Zia-Chahabi S., Zuber B., Tcherakian C., Farfour E., Vasse M. (2021). ADAMTS 13 deficiency is associated with abnormal distribution of von Willebrand factor multimers in patients with COVID-19. Thromb. Res..

[71] De Bruijn S., Maes M.B., De Waele L., Vanhoorelbeke K., Gadisseur A. (2021). First report of a de novo iTTP episode associated with an mRNA-based anti-COVID-19 vaccination. J. Thromb. Haemost..

[72] Yocum A., Simon E.L. (2021). Thrombotic Thrombocytopenic Purpura after Ad26.COV2-S Vaccination. Am. J. Emerg Med..

[73] Albánez S., Ogiwara K., Michels A., Hopman W., Grabell J., James P., Lillicrap D. (2016). Aging and ABO blood type influence von Willebrand factor and factor VIII levels through interrelated mechanisms. J. Thromb. Haemost..

